# Diagnostic scanographique d’une hernie inguino-scrotale de la vessie à propos d’un cas

**DOI:** 10.11604/pamj.2016.25.126.10280

**Published:** 2016-11-01

**Authors:** Nfally Badji, Hamidou Deme, Geraud Akpo, Mouhamadou Toure, Boucar Ndong, El Hadji Niang

**Affiliations:** 1 Service de Radiologie Générale, Hôpital Aristide Le Dantect, Dakar,Sénégal

**Keywords:** Vessie, hernie inguino-scrotale, TDM, Bladder, inguinoscrotal hernia, CT scan

## Abstract

We report the case of a patient aged 67 with a history of inguinal hernia, which featured a large painless purse evolving for several months associated with urinary disorders like urinary frequency. Ultrasound allowed highlight an emptiness of bladder lodge, urinary stasis and fluid collection in the scrotum that was mentioned a hydrocele. The abdominal pelvic CT revealed a bladder intra scrotal right situation associated with an inguinal hernia and direct left to bilateral urinary stasis. The diagnosis was confirmed by surgical exploration. The postoperative course was uneventful. The inguinoscrotal hernia exclusively bladder content is an exceptional entity. CT should be sought before any inguinoscrotal hernia associated with urinary disorders ( Mery 's Sign ).

## Abstract (en)

We report the case of a 67-year old patient with a personal history of inguinal hernia, presenting with a large painless bag associated with urinary disorders, similar to pollakiuria, evolving for several months. Ultrasound allowed to reveal an emptiness in the bladder bag, urinary stasis and a collection of fluid in the scrotum, evoking a hydrocele. Abdominal/pelvic CT scan showed right intrascrotal bladder associated with left direct inguinal hernia and bilateral urinary stasis. The diagnosis was confirmed by surgical exploration. The postoperative course was uneventful. Inguinoscrotal hernia containing exclusively urinary bladder is an exceptional entity. CT scan should be recommended for patients with inguinoscrotal hernia associated with urinary disorders (Mery's Sign).

## Introduction

La pathologie herniaire constitue la première intervention en chirurgie digestive. La vessie représente rarement le contenu de la hernie. Il s'agit le plus souvent d'une incarcération d'un diverticule ou d'une partie de la vessie [1]. Souvent asymptomatique le diagnostic est fait en per ou en postopératoire suite à des complications. Nous rapportons un cas de hernie inguinoscrotale droite, à contenu exclusivement vésical dont le diagnostic a été confirmé à la tomodensitométrie (TDM).

## Patient et observation

**Figure 1:**
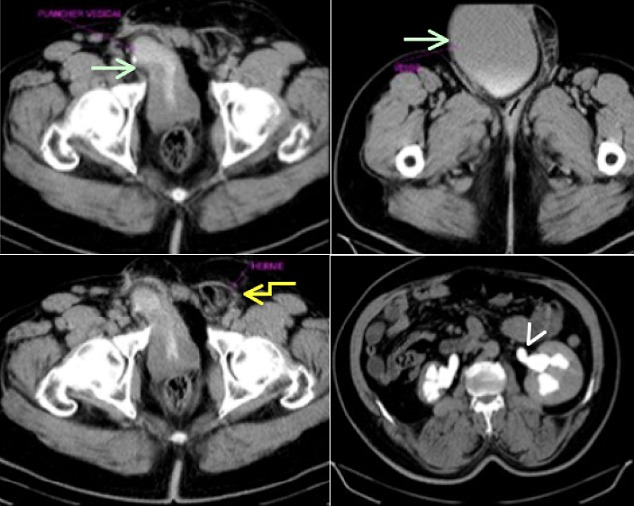
Uroscanner: coupes axiales TDM, montrant une hernie vésicale inguino-scrotale droite (flèche vert clair) et inguinale gauche (flèche jaune) avec une stase urinaire bilatérale (tête de flèche)

**Figure 2:**
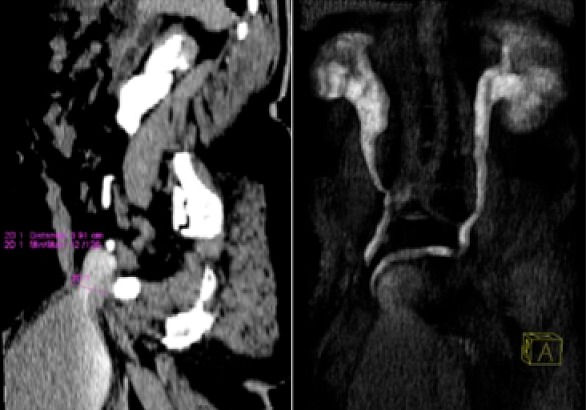
Uroscanner: reconstructions para sagittale droite et coronale en MIP montrant une hernie vésicale inguino-scrotale droite et une stase urinaire bilatérale

M.D.D âgé de 67 ans, aux antécédents de cure de hernie inguinale droite, reçu au service d'urologie pour une volumineuse bourse indolore évoluant depuis plusieurs mois associée à des troubles urinaires à type de pollakiurie. L'examen clinique a mis en évidence une cicatrice sur le pli inguinal droit. Le patient était apyrétique avec un état général conservé. Au toucher rectal la prostate était plate. Le bilan rénal était normal. L'échographie avait permis de mettre en évidence une vacuité de la loge vésicale, une collection liquide dans le scrotum et une stase urinaire bilatérale. Le scanner abdomino-pelvien (Figure 1, Figure 2) a mis en évidence une vessie en situation intrascrotale droite associée à une hernie inguinale gauche directe et à une stase urinaire bilatérale. L'exploration a été effectuée par voie inguinale bilatérale. Elle a visualisé la vessie dans la bourse droite. A gauche, on a noté une hernie inguinale directe. Les suites opératoires étaient simples.

## Discussion

Cette observation a montré la très bonne spécificité de la TDM pour le diagnostic de la hernie intra scrotale de la vessie. La tomodensitométrie après injection a montré à la phase de remplissage la localisation intra scrotale de la vessie qui a permis de poser de manière péremptoire le diagnostic. Les signes associés étaient la stase urinaire en amont qui montre le caractère compressif de cette pathologie menaçant ainsi la fonction rénale et la hernie inguinale directe gauche. Il aurait été difficile de poser le diagnostic si la vessie était vide. Dans lequel cas on aurait pu évoquer une hernie tissulaire, péritonéale en particulier. Dans la littérature cette pathologie représente 1 à 3% des hernies inguinales [2]. Elle se voit chez le sujet de plus de 50 ans. Il s'agit le plus souvent d'une incarcération d'un diverticule et ou d'une partie de la vessie dans le sac hernié. Ces formes sont asymptomatiques et le diagnostic se fait en per opératoire lors de la cure chirurgicale. La migration totale de la vessie dans le scrotum comme c'est le cas chez notre patient est qualifié d'exceptionnelle [3]. Le diagnostic est suspecté devant l'association d'une hernie inguino-scrotale et des signes urinaires et surtout d'une miction en deux temps avec la nécessité de comprimer le scrotum pendant la miction, « Mery's Sign » [4]. Il s'agit d'une pathologie dont le diagnostic est posé le plus souvent en peropératoire [5, 6]. Dans notre cas tous les signes cliniques évoqués dans la littérature ont été retrouvés. Les antécédents de cure de hernie constitueraient le facteur favorisant [7], cela a été retrouvé chez notre patient. Ce tableau clinique a permis dans notre observation d'évoquer le diagnostic et de poser l'indication d'un examen scanographique. L'exploration échographique a été effectuée chez ce patient avant l'examen scanographique. Elle a montré deux signes majeurs, la présence de liquide dans le scrotum qui faisait évoquer le diagnostic d'hydrocèle d'une part et d'autre part l'absence de vessie malgré la sensation impérieuse d'uriner du patient a qui on a fait ingérer de l'eau. Ces deux signes conjointement à un antécédent de récidive d'hernie auraient suffit pour étayer les bases du diagnostic. Certains auteurs soulignent les difficultés du diagnostic échographique dans les formes bilatérales [8]. En réalité le scanner à facilité le diagnostic en mettent en évidence un sac contenant de l'iode dans le scrotum. Le scanner a été d'une grande aide et a permis de consolider le diagnostic en montrant une triade très significative regroupant une envie impérieuse d'uriner, une vacuité vésicale et la présence de collection liquide entourée d'une paroi. Cette démarche prospective a permis de limiter les risques de lésions iatrogènes de la vessie au cours de l'intervention chirurgicale. L'urètrocystographie rétrograde (UCR) serait le meilleur examen dans l'exploration de la hernie vésicale. C'est l'examen qui devrait être demandé en première intention, du fait de sa disponibilité, de sa réalisation facile, rapide et de l'absence de contre-indication. Elle suffit pour poser le diagnostic et permettrait de faire le suivi post opératoire [9]. Cependant, elle occulte la stase urinaire haute. Chez notre patient, l'UCR n'a pas était demandée Cette observation nous suggère une attitude pratique bien différente de celle proposée par IZES Ba et al [10]. L'échographie abdomino-pelvienne devrait être de première intention et suffirait pour poser le diagnostic devant les signes cardinaux que sont l'envie impérieuse d'uriner, la vacuité de la loge vésicale, la présence de sac dans le scrotum. Ces signes auraient d'autant plus de valeur dans ce contexte de récidive de hernie inguinoscrotale. Le traitement se résume à la cure chirurgicale de la hernie avec réintégration de la vessie comme ce fut le cas chez notre patient. Les suites post opératoires étaient simples. L'UCR de contrôle était normale.

## Conclusion

La hernie vésicale inguinoscrotale est une entité exceptionnelle et survient le plus souvent chez un sujet de plus de 50 ans ayant des antécédents de cure de hernie inguinale. La symptomatologie est non spécifique, le diagnostic est suspecté par l'association de troubles urinaires ( Mery's Sign ) et confirmé par la tomodensitométrie.
